# Estimating Brain Load from the EEG

**DOI:** 10.1100/tsw.2009.83

**Published:** 2009-07-14

**Authors:** Anu Holm, Kristian Lukander, Jussi Korpela, Mikael Sallinen, Kiti M. I. Müller

**Affiliations:** ^1^ Brain Work Research Centre, Finnish Institute of Occupational Health, Helsinki, Finland; ^2^ Agora Center, University of Jyväskylä, Finland

**Keywords:** EEG, cognitive, workload, work load, brain, assessment of workload

## Abstract

Modern work requires cognitively demanding multitasking and the need for sustained vigilance, which may result in work-related stress and may increase the possibility of human error. Objective methods for estimating cognitive overload and mental fatigue of the brain on-line, during work performance, are needed. We present a two-channel electroencephalography (EEG)–based index, theta Fz/alpha Pz ratio, potentially implementable into a compact wearable device. The index reacts to both acute external and cumulative internal load. The index increased with the number of tasks to be performed concurrently (*p* = 0.004) and with increased time awake, both after normal sleep (*p* = 0.002) and sleep restriction (*p* = 0.004). Moreover, the increase of the index was more pronounced in the afternoon after sleep restriction (*p* = 0.006). As a measure of brain state and its dynamics, the index can be considered equivalent to the heartbeat, an indicator of the cardiovascular state, thus inspiring the name "brainbeat".

### INTRODUCTION

Modern working environments are often information intensive and work performance requires acting on multiple tasks simultaneously, i.e., multitasking. This is also true for everyday activities. For example, while driving a car, the driver can simultaneously navigate with the GPS and talk on the phone.

In a 24/7 society, shift work has increased in most professions[1], raising the risk of sleep deprivation. Multitasking requires contributions from prefrontal cortical regions that control attention functions. These prefrontal regions are also susceptible to sleep restriction[2,3,4]. The lack of sleep results in a nonoptimal physiological state for performing challenging tasks.

In many cases, these components of load, increased external task demands, and decreased internal physiological resources are present at the same time. Subjects suffering from even modest sleep loss have shown decreased performance in tasks that require neural control by prefrontal areas[5,6]. Warm et al.[7] and Young and Stanton[8] also raised the issue of mental underload and decreased vigilance, which can be as detrimental to performance as mental overload.

Predicting these performance decreases is difficult from performance data alone, since humans are able to maintain acceptable performance levels even with increasing task demands or under growing sleep pressure, to a certain point. Humans are also poor in self-identification of decreased vigilance and cognitive overload[5,6], which increases the risk of human error at work. If the state of increased workload could be detected, it would offer the possibility to either warn or assist subjects with automated systems before unfavorable performance failures occur.

Despite the fact that sustained high cognitive effort may have detrimental health effects and raises the risk of human error, studies on performance have typically focused on the efficacy of behavior, i.e., how well task demands are achieved. The physiological "costs" for maintaining the required performance level in demanding task conditions has been studied to a lesser degree. Maintaining acceptable performance levels under increased sleepiness or cognitive demands requires the mobilization of further brain resources, which is revealed as increased activation of physiological systems and can be detected by observing brain oscillations.

Brain oscillations are the biophysical result of complex interactions of neuronal networks, taking place both in the idling, as well as in the performing, human brain[9,10]. Oscillations of different frequencies have been linked to different functions of the brain. In electroencephalography (EEG) studies, growing task demands, as well as time-on-task demands, increase frontal theta activity and decrease parietal alpha activity[11,12,13,14,15,16]. Additionally, tasks that place extensive demands on executive functions affect frontoparietal EEG coherence in the alpha and theta bands[15,17]. The need for sleep (sleep pressure) can also be detected from the EEG spectrum. In awake subjects that are suffering from sleep deprivation, low-frequency EEG activity is increased in frontal areas with time awake, with only little circadian modulation[3,4]. A similar effect can also be seen during non-REM sleep[3].

Another EEG measure, the event-related potentials (ERPs), are neural responses to specific sensory and cognitive events. Especially, the P300 component of ERPs has been shown to be sensitive to the available processing capacity[18] and is thus widely used in cognitive workload assessment[19,20,21,22]. Despite the fact that ERPs provide a millisecond resolution for observing the subjects' cognitive processing, the methodology is not usable outside of the laboratory, as recording ERPs requires external stimuli, time locked to the measurement system, making the setup unrealistic for use during everyday activities.

EEG has the potential to identify changes in cognitive load in tasks that require continuous and intensive allocation of attention. Modern, compact measurement technologies enable measuring EEG even wirelessly during typical daily activities[23]. EEG measurements have adequate time resolution, conveying information nearly on-line. EEG thus provides a promising tool for assessing cognitive workload, comparable in simplicity to measuring the physical workload with heart rate monitors or pedometers.

We investigated how information derived from the EEG spectrum, especially frontal theta and parietal alpha activity, could be used as an indicator of overall brain load. This was done by combining EEG recordings with cognitive task performance, and manipulating external and internal factors affecting the person being tested.

### MATERIAL AND METHODS

#### Subjects

Twenty nonsmoking healthy men (19–22 years of age) participated in the changed task demands (CTD) condition and 16 participated in the time awake (TA) condition. The protocol was accepted by the local ethics committee. Informed consent was obtained from the subjects. Prior to the experiment, their health status was examined by a physician. The use of alcohol, tobacco, or caffeine was forbidden as of 24 h prior to the experiment. Each participant visited the laboratory three times: first they trained in the tasks until a constant performance level was reached with an individual task difficulty level[6]. On the second and third visits, the subjects performed the CTD and TA conditions.

On the first visit to the laboratory, each participant was given a sleep diary and was instructed to record information about their sleep for the 2 days prior to the forthcoming laboratory visits. The mean sleep length of the night immediately before the first visit was 408 ± 83 min (S.D.) and 505 ± 112 min (S.D.) the previous night. The corresponding values prior to the second visit were 425 ± 81 min (S.D.) and 488 ± 115 min (S.D.).

### Task

We used a modification of our computerized multitask[6], which is composed of four selectable subtasks (Fig. 1)**.** The auditory attention task consists of two tones (target and nontarget standard, 20 and 80% of tones, respectively, interstimulus interval 1.5 sec) delivered through loudspeakers. The subject presses a separate response pad when a target tone occurs. In the arithmetic task, subjects add numbers and input the answers with a mouse on a number pad presented on the monitor. In the memory task, the subjects detect whether the letter shown was among a list memorized before each trial. In the visual vigilance task, a dot appears in the center of the circle and starts to move towards the outermost border of the circle. The subject has to return the dot back to the innermost circle with a button press before the dot reaches the edge of the outermost circle. Subjects scored points with correct responses, and lost points with wrong or missed responses.

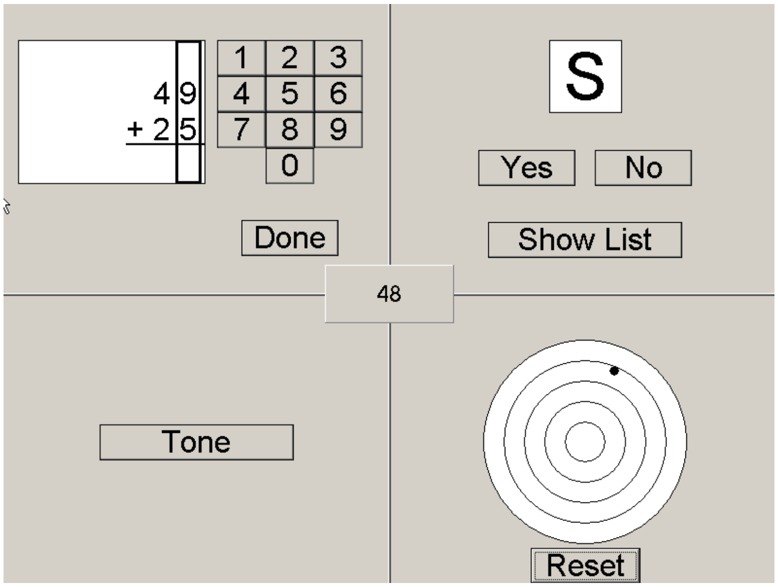

**FIGURE 1.** Example of the user interface of the computerized multitask. Top left, the arithmetic subtask; top right, the memory task; bottom left, the auditory attention task; bottom right, the visual vigilance task.

In the CTD condition, we varied the number of tasks the subjects performed simultaneously between single (auditory attention task), dual (auditory attention and arithmetic tasks), and multi (all four tasks simultaneously) conditions starting at 15:30. Each condition was done twice in counterbalanced order to reduce the time-on-task effect.

In the TA condition, we studied whether the EEG content could also be used to identify changes in internal load. We kept the multi condition demands constant and used time awake and sleep restriction as internal loading factors inducing sleep pressure. The subjects were instructed to perform all the subtasks equally well and as well as possible. In the normal sleep protocol, the subjects slept an 8-h night (23:00–07:00) in the laboratory. The following day, they performed four multi sessions, each lasting 70 min, with simultaneous EEG recording. Test sessions started at 8:30, 11:00, 13:30, and 15:45 (Fig. 2B). In the sleep

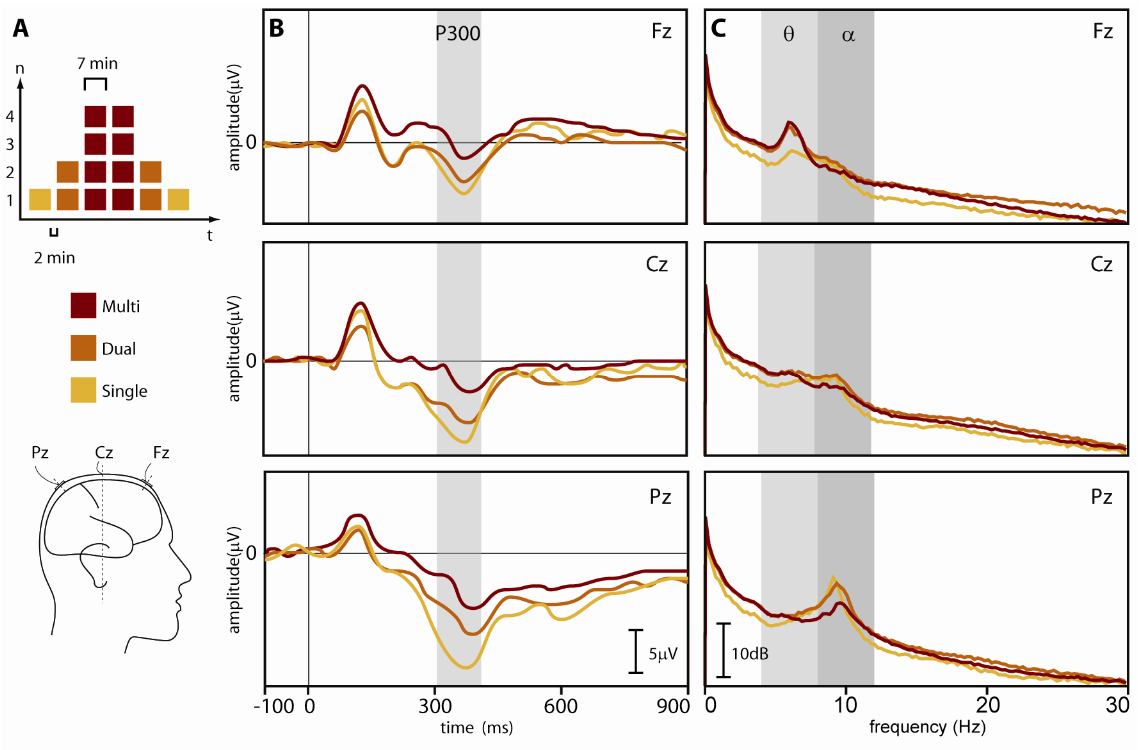

**FIGURE 2.** EEG spectrum is modulated by external demands. (A) Task setup. In the single condition, subjects performed an auditory attention task; in the dual condition, the auditory task together with an arithmetic task; and in the multi condition, four tasks (auditory, arithmetic, memory, and visual) concurrently. (B) Auditory ERPs in different task conditions. Increasing task demands decreased the P300 amplitude in the parietal area Pz ($F[1.5, 23.4] = 28.2$, $p = 0.001$, $n = 20$). Negative plotted upwards. (C) EEG spectra. Increasing task demands increase frontal theta (Fz) and decrease parietal alpha (Pz). Only the change in alpha range is statistically significant.

restriction protocol, the subjects slept 2 h between 05:00–07:00 in the laboratory and performed four multitask sessions similar to the normal sleep protocol.

### EEG Measurements

EEG was measured continuously with silver electrodes, referenced to the right and grounded to the left mastoid (impedance <5 kΩ ), pass-band filtered (0.5–50 Hz), and digitized at 500 Hz using a SynAmps amplifier. With Scan Edit 4.3.3 utility, we transformed the data measured to 4-sec epochs, corrected for eye movement artefacts with the ocular artefact reduction (OAR) utility, excluded epochs containing other artefacts (±70 μV), and computed spectrograms for the 10–20 system derivations Fz, Cz, and Pz using a Fast Fourier transformation. The sweeps were smoothed using a 2048-sample Hanning window, and absolute spectral power values for theta (4–8 Hz) and alpha (8–12 Hz) were calculated.

### ERP Measurements

For P300 analysis, EEG was transformed to epochs of –100 to 900 msec relative to the onset of the target tones in the auditory attention task. Eye movements were corrected and artefacts removed as in the EEG analysis. The response for auditory target tones was computed for the 10–20 system derivations Fz, Cz, and Pz. The average waveforms were low-pass filtered at 20 Hz, and P300 component detected as a maximum positive peak within 250–550 msec. The amplitude of P300 was measured relative to the 100-msec prestimulus baseline.

### Statistical Analyses

Statistical analyses were performed using repeated measures of ANOVA in SPSS 12 for Windows. When studying the power spectrum values, the data were log transformed to achieve normal distribution. Greenhouse-Geisser degrees of freedom were used to correct the violations of the spherical assumption when appropriate. To explore and compare metrics that correlated with the changes of brain loads quantitatively, the Receiver Operating Characteristic (ROC) curve analysis was carried out. For this purpose, the difference between each parameter and the baseline value (auditory oddball condition) was calculated and used in the ROC analysis. To study the effect of the sleep deprivation on performance and EEG content, corresponding values in the sleep restriction protocol and the normal sleep protocol were subtracted from each other. The significance of the sleep restriction effect was studied with Wilcoxon's signed rank test.

## RESULTS

### EEG Spectrum and External Factors

To determine the effects of external load on the EEG spectrum, we varied the number of tasks the subjects performed simultaneously between single (one task), dual (two tasks), and multi (four tasks) conditions (Fig. 2A). To verify that the increase in the number of tasks to be performed concurrently actually increased the cognitive workload of the subjects' brain, we measured ERPs to the auditory attention task simultaneously with other EEG measures. In accordance with a previous report[18], the P300 amplitude of ERP decreased with increasing task demands (Fig. 2B), showing that our loading manipulation was successful.

The absolute EEG power spectrums for the different task conditions are shown in Fig. 2C. A growth in task demands increased the amount of theta activity in the frontal site (Fz) and decreased alpha activity in the parietal site (Pz), as was also reported in previous studies[11,12,13,14,16]. However, only the change in the alpha range was statistically significant.

Even though the P300 amplitude decrease shows that the cognitive workload increases, the absolute power of the EEG spectrum ranges themselves were not sensitive enough to detect this change. However, the visual inspection of the data in Fig. 2C revealed that theta increases and alpha decreases with increasing task difficulty. The trends seen inspired us to test whether we could improve the estimation of brain load by calculating the ratio of the absolute power of frontal theta activity to the absolute power of parietal alpha activity (theta Fz/alpha Pz). We discovered that the value of this index increased with increasing cognitive task demands ($F[1.3, 25.0] = 8.6$, $p = 0.004$, n = 20; single vs. dual, $p = 0.002$; dual vs. multi, $p = 0.014$) (Fig. 3A). The index value was already higher in the dual condition in 15/20 and almost in all cases in the multi condition (19/20) when compared to the index value in the single condition.

The comparison of the brain load metrics with ROC curves revealed that the ratio performed well in brain load estimation when compared to the P300 amplitude or the power measures (Fig. 4 and Table 1).

We also tested whether different combinations of the theta and alpha power (i.e., theta Fz * alpha Pz, log [theta Fz]/log [alpha Pz], or theta Fz + alpha Pz) could differentiate brain load levels. The ROC analysis showed that these combinations performed at the level of the parietal alpha power, but did not reach the level of the theta Fz/alpha Pz ratio (Table 1).

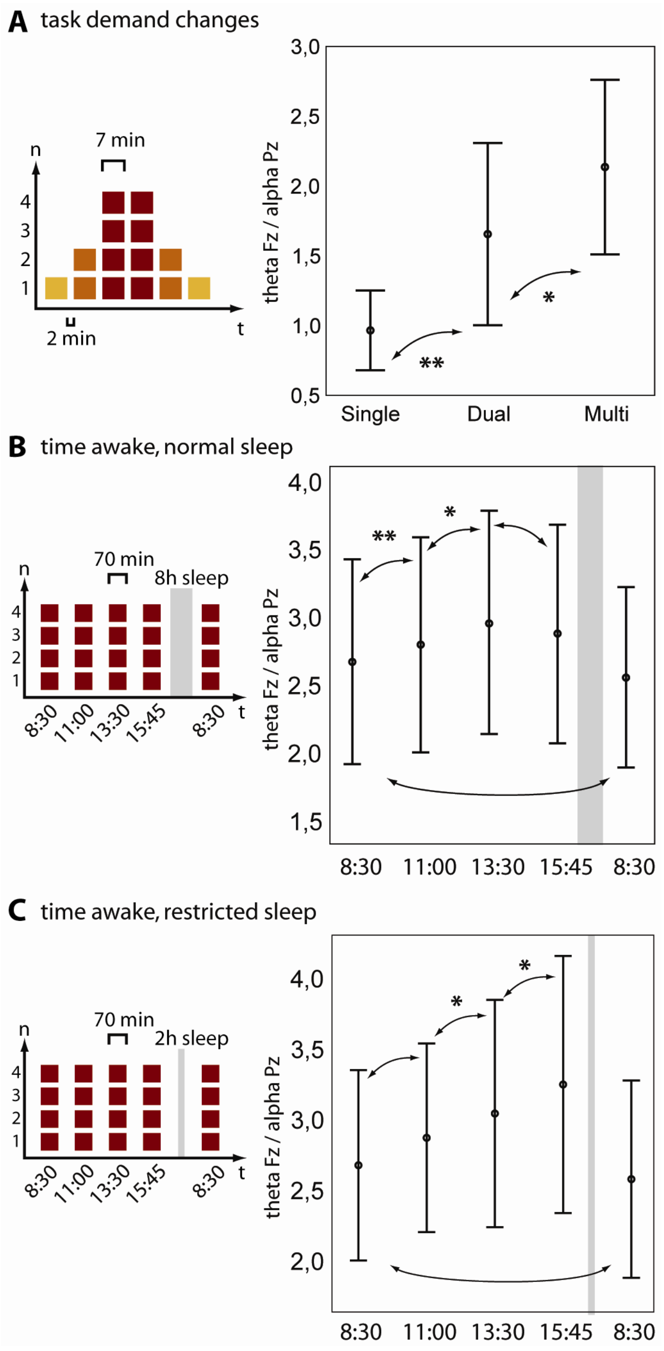

**FIGURE 3.** Theta Fz/alpha Pz correlates with external and internal factors. (A) The theta Fz/alpha Pz ratio increased systematically with increasing task demands (F[1.3, 25.0] = 8.6, $p$ = 0.004, n = 20; single vs. dual, $p$ = 0.002; dual vs. multi, $p$ = 0.014). (B) Normal sleep protocol. The theta Fz/alpha Pz ratio increased with time awake (F[2.4, 35.6] = 7.1, $p$ = 0.002, n = 16; 8:30 vs. 11:30, $p$ = 0.009; 11:30 vs. 13:30, $p$ = 0.029; 13:30 vs. 15:45, $p$ = 0.168). After a well-slept night, the ratio returned to the baseline value, $p$ = 0.139, even though the performance was improved. (C) Sleep restriction protocol. The theta Fz/alpha Pz ratio increased with time awake (F[2.3, 34.0] = 6.2, $p$ = 0.004, n = 16; 8:30 vs. 11:00, $p$ = 0.096; 11:00 vs. 13:30, $p$ = 0.027; 13:30 vs. 15:45, $p$ = 0.017). After a well-slept night, the ratio returned to the baseline value, $p$ = 0.477. Error bars indicate S.E.M.

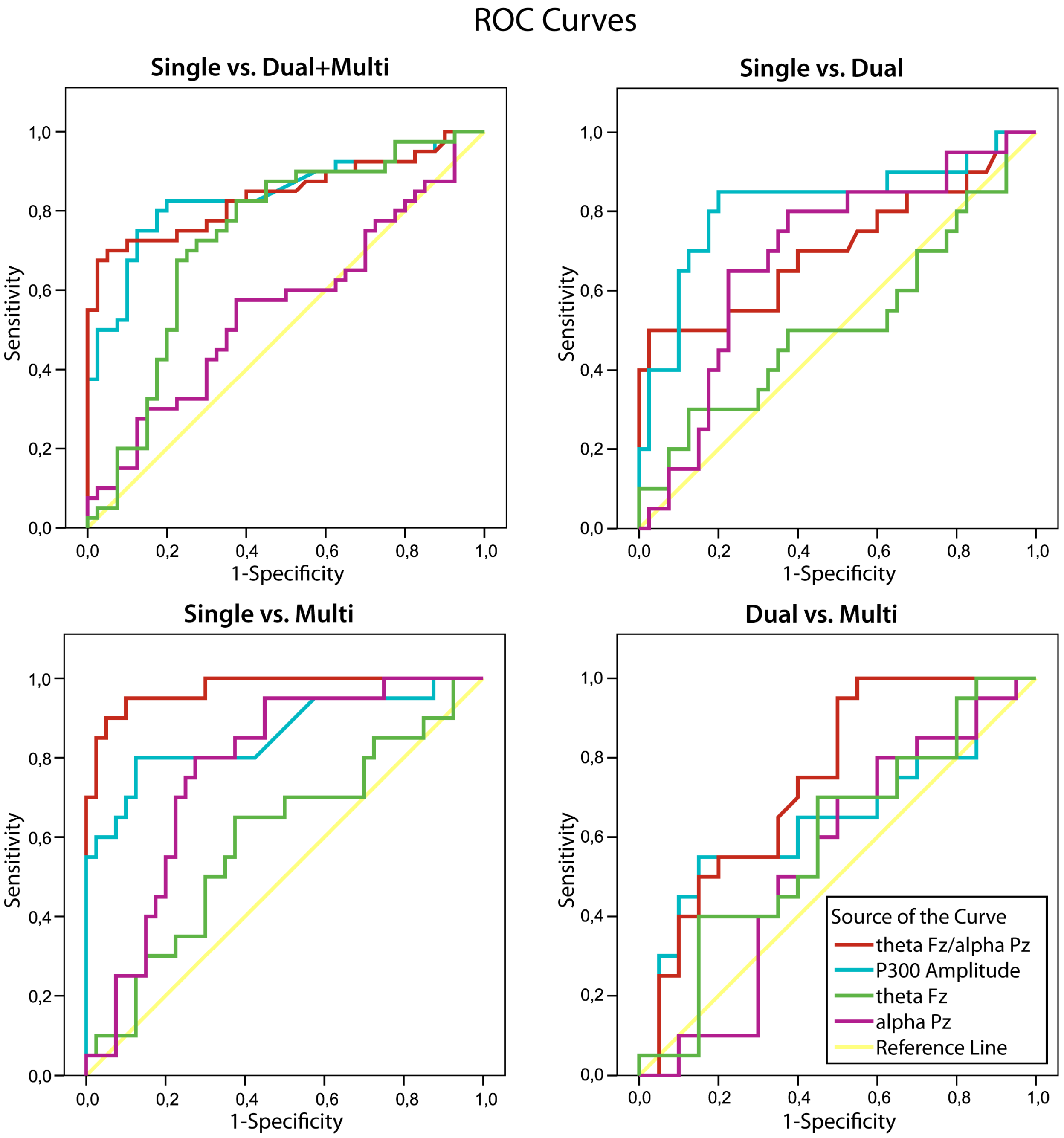

**FIGURE 4.** ROC curve comparisons of different brain metrics parameters**.** Area under a ROC curve is plotted for single vs. dual (upper left), single vs. multi (upper right), dual vs. multi (lower left), and single vs. dual + multi (lower right) situations. The P300 amplitude and theta Fz/alpha Pz ratio perform equally well in discriminating task demands. The alpha power also performs reasonably well, where as the theta power does not.

**TABLE 1**
**The Comparison of Different Brain Load Metrics***

| Parameter | Area Under Curve | | | |
|---|---|---|---|---|
| | Single vs. Dual + Multi | Single vs. Dual | Single vs. Multi | Dual vs. Multi |
| P300 amplitude | 0.84 ($p < 0.001$) | 0.82 ($p < 0.001$) | 0.86 ($p < 0.001$) | 0.64 ($p = 0.123$) |
| Theta Fz/alpha Pz | 0.84 ($p < 0.001$) | 0.71 ($p = 0.008$) | 0.97 ($p < 0.001$) | 0.75 ($p = 0.008$) |
| Theta | 0.56 ($p = 0.376$) | 0.52 ($p = 0.802$) | 0.59 ($p = 0.233$) | 0.54 ($p = 0.646$) |
| Alpha | 0.73 ($p < 0.001$) | 0.69 ($p = 0.016$) | 0.77 ($p = 0.001$) | 0.60 ($p = 0.317$) |
| Theta Fz + alpha Pz | 0.74 ($p < 0.001$) | 0.69 ($p = 0.017$) | 0.79 ($p < 0.001$) | 0.59 ($p = 0.358$) |
| Theta Fz * alpha Pz | 0.64 ($p = 0.031$) | 0.60 ($p = 0.198$) | 0.68 ($p = 0.026$) | 0.54 ($p = 0.665$) |
| lg(Theta Fz)/lg (alpha Pz) | 0.72 ($p = 0.001$) | 0.67 ($p = 0.037$) | 0.77 ($p = 0.001$) | 0.62 ($p = 0.194$) |

* ROC curve was used to compare the classification performance of brain load metrics P300 amplitude, theta Fz/alpha Pz ratio, theta and alpha power, theta power + alpha power, theta power * alpha power, and lg(theta Fz)/lg (alpha Pz) ratio. Area under ROC curve is shown with *p* value.

### Internal Load Affects EEG Spectrum

#### *Normal Sleep*

After normal sleep, time awake had a statistically significant effect on both the frontal theta and the parietal alpha, F(1.382, 20.724) = 6.082, $p = 0.015$ and F(1.999, 29.978) = 4.365, $p = 0.022$, respectively. The power of frontal theta activity increased with increasing time awake (8.30 vs. 11.15, $p = 0.004$; 11.15 vs. 13.30, $p = 0.051$; 13.30 vs. 15.45, $p = 0.071$). The power of parietal alpha activity also increased somewhat with increased time awake (8.30 vs. 11.15, $p = 0.056$; 11.15 vs. 13.30, $p = 0.036$; 13.30 vs. 15.45, $p = 0.252$). The recovery night restored the absolute power of both theta and alpha activity to the baseline levels, $p = 0.211$ and $p = 0.372$, respectively.

Performance in the multitask was stable across all four sessions (F[1.87, 28.06] = 2.276, $p = 0.124$), while the theta Fz/alpha Pz ratio increased with time awake (F[2.4, 35.6] = 7.1, $p = 0.002$, n = 16; 8:30 vs. 11:30, $p = 0.009$; 11:30 vs. 13:30, $p = 0.029$; 13:30 vs. 15:45, $p = 0.168$), (Fig. 3B). Additionally, to test the effect of a well-slept night (8 h) on theta Fz/alpha Pz ratio, a fifth test session was carried out at 8.30 a.m. the next morning (Fig. 3B). The performance after the second night was better than during the sessions on the previous day ($p < 0.05$ in all comparisons) and the theta Fz/alpha Pz ratio recovered to the value of the previous morning.

#### *Sleep Restriction*

When the subjects were sleep deprived, performance in the multitask deteriorated across the four sessions (F[2.34, 23.26] = 6.68, $p = 0.004$; 8:30 vs. 11:00, $p = 0.234$; 11:00 vs. 13:30, $p = 0.051$; 13:30 vs. 15:45, $p = 0.032$). The theta Fz/alpha Pz ratio increased with time awake (F[2.3, 34.0] = 6.2, $p = 0.004$, n = 16; 8:30 vs. 11:00, $p = 0.096$; 11:00 vs. 13:30, $p = 0.027$; 13:30 vs. 15:45, $p = 0.017$) (Fig. 3C).

The performance after the second night (8-h sleep) was better than during the session at the same time on the previous morning after 2-h sleep ($p < 0.001$). The theta Fz/alpha Pz ratio recovered to the value of previous morning, $p = 0.477$.

### Difference between Normal Sleep and Sleep Restriction

The performance in the multitask was significantly decreased after sleep deprivation when compared to the performance in the normal sleep protocol at the same time of day. The performance difference between sleep deprivation and normal sleep state was statistically significant in all four comparisons: at 8:30, $p = 0.002$; at 11:00, $p = 0.004$; at 13:30, $p = 0.008$; at 15:45, $p = 0.001$. The theta Fz/alpha Pz ratio was larger after sleep restriction than after normal sleep: at 15:45, $p = 0.006$. After the 8-h recovery night, the theta Fz/alpha Pz ratio did not differ from the normal sleep protocol: at 8:30, $p = 0.393$. On the contrary, performance after the 2- and 8-h nights was lower than after two 8-h nights in the normal sleep protocol ($p = 0.036$), and was comparable to the performance level after one 8-h night after the normal sleep protocol ($p = 0.681$).

### Estimating Workload On-Line with EEG

Thus far, we have shown that the theta Fz/alpha Pz ratio indicates changes in the internal and external load on group level. For the ratio to function as a continuous monitor of brain load in everyday use, the index should be reactive enough to reflect temporary changes in cognitive task demands. To test this, we introduced task demand changes to a few subjects continuously, without pauses between sessions. The preliminary data for three single subjects presented in Fig. 5 show that the changes in task demands result in rapid changes in the values, especially when comparing the switch from, cognitively, the least demanding single to either the dual or multi conditions.

## DISCUSSION

Here we have reported the effects of internal and external load on EEG content. We manipulated external load by changing the number of tasks to be performed simultaneously. Internal load was manipulated with time spent awake and sleep restriction. We have shown that the frontal theta/parietal alpha ratio is sensitive to these manipulations and increases both with growing task demands and time spent awake. This study extends the previous findings on EEG content in awake, task-performing subjects, by taking into account the interactions of frontal and parietal areas during challenging task performance, and providing a tool for overall brain load assessment.

We have shown that the theta Fz/alpha Pz ratio was more sensitive in detecting increased task demands than the absolute power values of frontal theta and parietal alpha, which have been shown to be reactive to task demand manipulations[11,12,13,14,15,16]. However, absolute power values have not been sensitive enough to be used in continuous monitoring of brain load in everyday use. Therefore, their sensitivity has been improved with advanced analysis methods[14,24,25] and combined analysis of other physiological measures[26]. We suggest that the simple theta Fz/alpha Pz ratio described in our paper is, as such, sensitive enough to detect workload changes.

The increase in the theta Fz/alpha Pz ratio with time awake, both after normal 8-h sleep and restricted 2-h sleep, and an enhancement of the time awake effect by sleep restriction are in good accordance with previous findings that have shown that frontal theta increases with time awake, with minor circadian and major homeostatic modulation, whereas parietal alpha activity exhibited circadian variation and no increase with time spent awake[3,4]. In addition, high sleep pressure increased frontal theta and low sleep pressure increased parietal alpha in comparison to the baseline. Even though subjects in these previous studies were performing simpler tasks than our multitask, the results support the idea that the ratio represents the level of sleep pressure caused by time spent awake instead of pure circadian modulation.

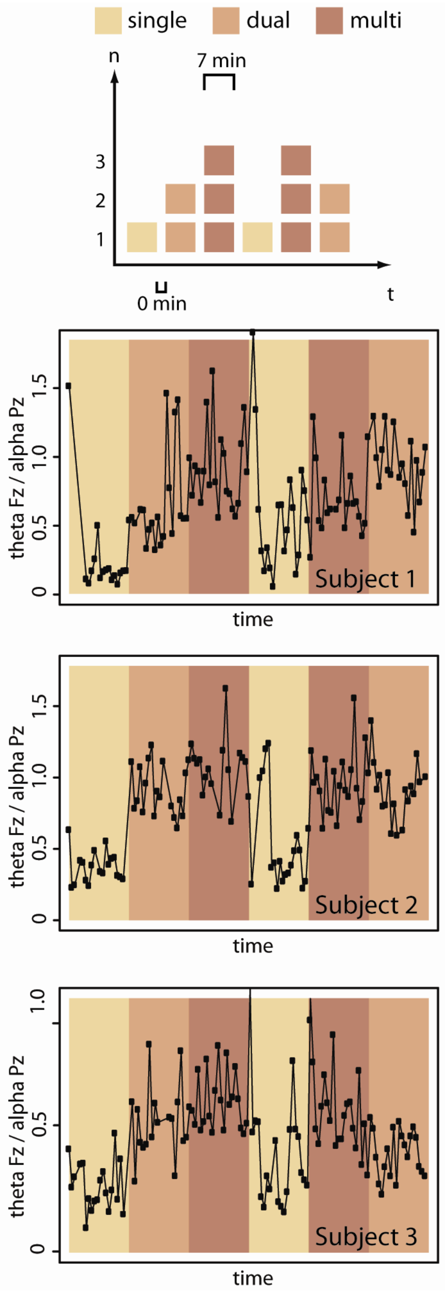

**FIGURE 5.** Examples of reactivity of the theta Fz/alpha Pz ratio to continuous changes in task demands. Task setting: the subjects performed the single, dual, and multi conditions continuously, 7 min per condition, for a total time of 42 min. The value is averaged over 20 sec. The change between single compared to dual and multi was clear, but between dual and multi at the individual level varied somewhat more (clear in subjects 2 and 3, but not in 1). The fluctuating attention and/or effort of the subject may affect the ratio.

We have also shown that while subjects' performance in the multitask was stable across four sessions performed during a single day in the laboratory, the theta Fz/alpha Pz ratio increased with increased time awake and sleep restriction enhanced the effect. This indicates that subjects had to put in more effort to sustain the performance level, which affected brain physiology. This is in accordance with the cognitive-energetical model described by Hockey[27], which states that performance may be protected under stress by the requirement of further resources, but only at the expense of increased subjective effort, and behavioral and psychological costs.

Our study showed that after two well-slept nights, performance was better than after one well-slept night, and the theta Fz/alpha Pz ratio recovered to the level of one well-slept night. On the contrary, after sleep restriction, the theta Fz/alpha Pz ratio recovered, whereas the performance did not show learning, but stayed at the same level as with one 8-h sleep. Sleep is considered essential for brain plasticity, restoration of energy resources, as well as consolidation of memory traces and learning[28,29]. Additionally, both increased sleep pressure and task demands have been shown to affect energy metabolism of the brain[27,30,31]. We suggest that consumption and restoration of energy resources are reflected in the theta Fz/alpha Pz ratio.

A somewhat similar approach, but in resting subjects, has been used in neurofeedback studies[32]. In these studies, theta/alpha ratio is measured using only a parietal electrode Pz, and feedback of the alpha and theta signal is given by auditory tones. In neurofeedback applications, this ratio has been used to produce a hypnagogic state, similar to a meditative or hypnotic state of relaxation, in an eyes-closed condition over a 30- or 40-min feedback session. Repeated sessions where subjects have drifted down to alpha-theta state have resulted in long-term abstinence and changes in personality testing results. In our study, we also tested whether workload and increasing sleep pressure could be detected on alert subjects, who simultaneously perform a challenging task, using only the parietal theta Pz/alpha Pz ratio. We discovered that while the ratio somewhat reacts to workload manipulations, it was not as sensitive as an index where frontoparietal information is used, i.e., when the ratio is calculated as theta Fz/alpha Pz (sensitivity for dual vs. single condition, 0.6 for theta Pz/alpha Pz and 0.75 for theta Fz/alpha Pz; for multi vs. single, 0.8 for theta Pz/alpha Pz and 0.95 for theta Fz/alpha Pz).

In addition to neurofeedback studies, the use of the frontal theta/parietal alpha ratio in a task load estimation has been suggested in Postma et al.[33]. Contrary to their findings, we also found that the internal state of the subject has a strong effect on the ratio. Time awake significantly increased the theta Fz/alpha Pz ratio. Moreover, we found that sleep restriction enhanced this increase.

Lower frequency oscillations (delta, theta, and alpha) have been suggested to form the basis for longer-range communication between brain areas[9,34]. Especially, increased synchronization in lower frequencies between posterior and frontal regions during cognitive task performance has been shown with EEG studies[15,35]. In addition to task demand changes, frontal low frequency activity has been connected to sleep pressure level in the awake subject[4]. We suggest that the theta Fz/alpha Pz ratio measures the overall brain load (a sum of internal and external factors affecting the brain) at a given time. Our study showed that the frontal theta increased and the parietal alpha decreased as the task load increased, whereas both variables increased as time awake increased. These two loading factors seem to have partly different neural origin, as has also been reported in a functional magnetic resonance imaging study by Drummond et al.[36]. However, both increased external and internal load increase the theta Fz/alpha Pz ratio, suggesting that it may be used in overall brain load estimation. Because the index reacts to loading changes of the brain, we named it "brainbeat".

Quantitative comparison of the P300 amplitude, theta Fz/alpha Pz ratio, theta and alpha power parameters with ROC curves showed that the ratio performed well in differentiating task demand levels. The ratio was comparable with the P300 amplitude, making it possible to estimate the task demand levels without an additional oddball task required by the P300 measure.

Our study also showed that the alpha power could differentiate task demand levels from each other. However, alpha power is not an optimal method for estimating overall brain load, since it increases both when an alert subject is working at easy task demand level, as well as when a subject is engaged with complex multitask and sleep pressure increases.

The effects of internal and external factors cannot be distinguished from one another solely on the basis of the brainbeat value. On the contrary, we hypothesize that the same level of brain load can be caused by different loading factors. Thus, for example, a high external load, such as demanding work, causes a temporary loading effect that is comparable to what would be accumulated by the gradual increase of the internal load as a result of time spent awake. It is also possible that when the internal state of the subject is not optimal, cognitive overload situations may develop faster in high workload conditions in relation to low workload conditions. This creates restrictions to the usability of the brainbeat in brain computer interaction applications, where internal and external load need to be differentiated. However, in many other applications, (e.g., in traffic safety issues), detecting overload situations regardless of their origin is essential in order to avoid detrimental errors.

Assessment of the brainbeat is based on information obtained with two EEG electrodes and can be implemented into a compact, wearable device for on-line monitoring of brain state in both field and clinical settings. It can be used as a first-stage diagnostic tool and follow-up measure of the overall functional state of the brain. This enables the study of the effects of various stressors on brain physiology in both daily life and at work. Such information could be used to ensure that operational environments at work and in everyday life are humane by, for example, optimizing cognitive demands and work shifts, and thus protecting the health of a “working brain”. Oscillatory phenomena of the neural networks can be seen as a metric for the functional state of the brain equivalent to the heartbeat as a metric of the physiological state of the cardiovascular system. As the brainbeat reacts to both acute external and cumulative internal load, we suggest that it could be used as a measure of the overall brain load.
